# Comparative study on pregnancy complications: PGT-A vs. IVF-ET with gender-specific outcomes

**DOI:** 10.3389/fendo.2024.1453083

**Published:** 2024-11-06

**Authors:** Ling Guo, Xiao Li, Anliang Guo, Yufeng Wang, Yue Liang, Yan Li, Xinbo Xu, Hong Lv

**Affiliations:** ^1^ Department of Obstetrics and Gynecology, Qilu Hospital of Shandong University, Jinan, Shandong, China; ^2^ Medical Integration and Practice Center, Shandong University, Jinan, Shandong, China; ^3^ State Key Laboratory of Reproductive Medicine and Offspring Health, Center for Reproductive Medicine, Institute of Women, Children and Reproductive Health, Shandong University, Jinan, Shandong, China; ^4^ Department of Otolaryngology, Qilu Hospital of Shandong University, Jinan, Shandong, China

**Keywords:** preimplantation genetic testing for aneuploidy, *in vitro* fertilization and embryo transfer, singleton pregnancy, fetal sex, preeclampsia

## Abstract

The safety and clinical effectiveness of preimplantation genetic testing for aneuploidy (PGT-A) in improving pregnancy outcomes for sub-fertile patients remains controversial. Potential sex-based differences in the relationship between PGT-A and pregnancy complications have not been investigated, which could guide the appropriate clinical application of PGT-A. In this secondary analysis of data from a multicenter, randomized, controlled, non-inferiority trial (NCT03118141), 940 women who achieved singleton live birth during the trial were included to estimate the between-group differences in pregnancy complications following PGT-A versus conventional *in vitro* fertilization (IVF) vary with fetal sex. Logistic regression analysis was used to adjust for possible confounders, and subgroup analysis was also performed. Among male fetuses, the risk of maternal preeclampsia was significantly lower after PGT-A compared to conventional IVF treatment (3.37% vs. 7.88%; adjusted OR, 0.40; 95% CI, 0.17-0.92; *P* = 0.032). However, this protective effect was not observed in pregnancies with female fetuses (3.63% vs. 3.38%; adjusted OR, 1.04; 95% CI, 0.36-3.00; *P* = 0.937). In addition, no significant sex-dependent differences in the risks of other pregnancy complications or neonatal outcomes were detected between PGT-A and conventional IVF groups (*P* > 0.05). In summary, PGT-A was associated with a decreased risk of maternal preeclampsia in singleton pregnancies with male fetuses, highlighting its potential utility in preeclampsia prevention in addition to spontaneous abortion rate reduction.

## Introduction

Preimplantation genetic testing for aneuploidy (PGT-A), a derivative technology of *in vitro* fertilization and embryo transfer (IVF-ET), has become increasingly utilized to improve outcomes in the field of assisted reproduction (1). The goal of PGT-A is to identify euploid embryos that are suitable for transfer, thereby increasing the live birth rates and reducing the risk of adverse pregnancy outcomes, such as spontaneous abortion (2). Some studies have shown that PGT-A can significantly improve pregnancy outcomes compared to conventional IVF, especially in women of advanced maternal age (3, 4). However, our recent randomized controlled study indicates that despite a perceived reduction in the frequency of pregnancy loss among clinical pregnancies for the PGT-A group, it does not positively affect cumulative live birth rates following embryo transfer in patients with a good prognosis, nor does it impact other obstetric pregnancy or neonatal outcomes (5). This provokes debates regarding whether the purported clinical effectiveness and benefits of PGT-A justify the risks intrinsic to the testing process. Despite controversy surrounding the invasiveness of this procedure for transferred embryos, PGT-A is currently utilized in approximately half of all IVF cycles in the USA, with its application steadily increasing worldwide (6). Thus, evidence regarding the effectiveness and safety of PGT-A is urgently needed for guiding the correct indications and optimal utilization.

Preeclampsia, a common pregnancy complication characterized by *de novo* development of high blood pressure (≥140/90 mm Hg) concurrent with proteinuria (≥300 mg/L per 24 hours) after 20 weeks of gestation, remains a leading cause of maternal and perinatal mortality and morbidity worldwide (7) and is associated with a substantial long-term risk for cardiovascular disease (8). One previous report showed a higher risk of preeclampsia associated with frozen single blastocyst transfer compared to fresh single blastocyst transfer (9). It is now broadly accepted that placental dysfunction, originating from the trophectoderm of the embryo, contributes to preeclampsia pathogenesis (7). Given that PGT-A necessitates biopsy of embryonic trophectoderm cells for genetic examination, it could provide valuable insights into preventing diseases related to embryonic trophectoderm abnormalities, such as preeclampsia. However, the accuracy and reliability of PGT-A results should be carefully validated due to potential confounding factors.

Furthermore, emerging evidence indicates that selecting euploid embryos via PGT-A may be misleading, as the genetic features of the trophectoderm do not fully represent the chromosomal status of the inner cell mass, which eventually develops into the fetus. Given the self-correction ability of aneuploid cells in the embryo and the presence of mosaic cells in the placenta of healthy pregnancies, as revealed in recent studies (6, 10, 11), PGT-A could potentially produce high false-positive rates for aneuploidy, resulting in a waste of transferrable embryos. Moreover, an increasing number of studies highlight that placenta formation and function during pregnancy are sex-dependent, and that male fetuses carry a higher risk of pregnancy complications, including gestational diabetes, gestational hypertension, preeclampsia, and eclampsia (12–14). However, studies investigating the effects of PGT-A on pregnancy complications, particularly those related to placental dysfunction, have generally disregarded differences in fetal sex (15).

Our group conducted a multicenter, randomized, controlled, non-inferiority trial and observed no improvement in the cumulative live birth rate by PGT-A compared to IVF in women with a good prognosis (5). In this study, we further performed a *post hoc* exploratory secondary analysis among women who achieved a singleton live birth during this trial and assessed the differences in pregnancy complications between male and female fetuses in PGT-A versus conventional IVF.

## Materials and methods

### Design and participants

**Figure 1:**
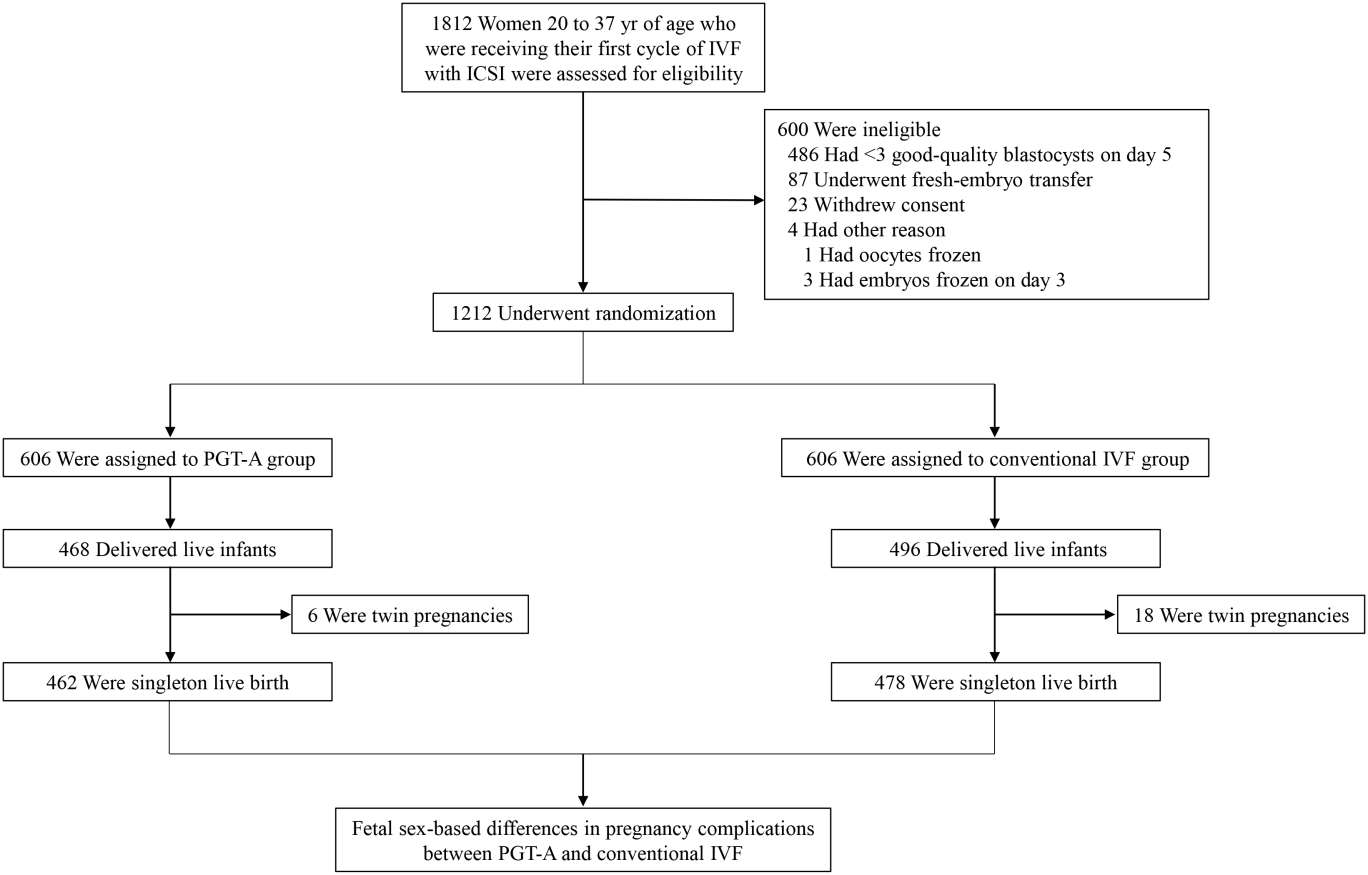
Flow chart of participants screening and enrollment. IVF, *in vitro* fertilization; ICSI, intracytoplasmic sperm injection; PGT-A, preimplantation genetic testing for aneuploidy.

The original multicenter, randomized, controlled clinical trial (registration number: NCT03118141) was conducted sequentially from July 2017 through June 2018, and the primary outcomes and protocols of the trial are published (5). Briefly, 1212 women who planned to undergo their first IVF cycle with a good prognosis for live birth were enrolled. A good prognosis was defined by patient age of 20 to 37 years and the availability of three or more high-quality blastocysts. All participants were randomized and then assigned to either the PGT-A group or the conventional IVF group (each group included 606 women). In the present analyses, the subjects without a live birth or with a multiple pregnancy were excluded, resulting in a total cohort of 940 women who achieved singleton live birth for further evaluation (**Figure 1**).

### Study procedure

Ovarian stimulation was performed through long or short regimens of gonadotropin-releasing hormone (GnRH) agonist or with a GnRH antagonist. The detailed schedule for controlled ovarian hyperstimulation has been previously described (5, 16). Human chorionic gonadotropin (hCG), GnRH agonist, or both were administered to trigger oocyte maturation when at least two follicles were ≥18 mm. All oocytes were inseminated by intracytoplasmic sperm injection (ICSI), and viable embryos were cultured to the blastocyst stage. According to the trial design, three high-quality blastocysts selected by means of morphological criteria (17) in the PGT-A group underwent trophectoderm biopsy. In each cycle, a euploid blastocyst in the PGT-A group or a high-quality blastocyst in the conventional IVF group was chosen for transfer. Endometrial preparation before frozen embryo transfer was performed with either a natural ovulation cycle, an artificial regimen, or an ovulation induction cycle. Luteal-phase support was administered in both groups. These techniques have been previously described in detail (16, 18). In this trial, up to three blastocyst-stage embryos were transferred either in the PGT-A group or conventional IVF group within 1 year after randomization until live birth or pregnancy termination. All pregnancy and neonatal outcomes were recorded in detail. Only singleton live births were included in the present study.

### Study outcomes

The primary outcomes were pregnancy complications, principally including the incidence of preeclampsia in subjects with singleton live births following PGT-A or conventional IVF. Secondary outcomes were neonatal outcomes, mainly including mean birth weight, as well as the incidence of small for gestational age (SGA) and large for gestational age (LGA) of singleton fetuses. The parameters of controlled ovarian hyperstimulation and other pregnancy complications were also analyzed. Preeclampsia was defined as hypertension appearing after 20 weeks of gestation with proteinuria (≥ 0.3 mg urinary protein per 24 h) (19, 20). SGA and LGA were defined as smaller than the 10th percentile or larger than the 90th percentile of birth weight at the corresponding gestational week, respectively, with consideration of fetal sex (21).

### Statistical analysis

Statistical analyses were performed using SPSS 26.0 for Windows (IBM, Armonk, NY, USA). The Kolmogorov–Smirnov test, in conjunction with histogram and Q-Q plot, was used to test for normality in the distribution of data. Continuous variables with normal distributions are presented as the mean ± standard deviation (SD) and were analyzed by independent *t test*. The median with interquartile range is used to show data with a non-normal distribution and compared by the Mann–Whitney U test. Categorical variables are presented as counts (percentages) and were compared using either the chi-square or Fisher’s exact test. Multivariable logistic regression analysis was performed to adjust for baseline characteristics. Multivariable linear regression or logistic regression analysis was used to investigate potential associations between PGT-A and pregnancy complications, adjusting for maternal age, body mass index (BMI), serum anti-Müllerian hormone (AMH) level, number of oocytes retrieved, number of high-quality embryos on day 5 or 6, endometrial thickness before embryo transfer, PGT-A vs. conventional IVF, fetal sex, and interaction between PGT-A vs. conventional IVF and fetal sex. Values of *P* < 0.05 were considered statistically significant.

## Results

### Baseline characteristics

This study included a total of 940 women who achieved singleton live birth after PGT-A or conventional IVF during the trial. Among them, 462 women were randomly assigned to the PGT-A group, and 478 women were assigned to the conventional IVF group. As shown in **Table 1**, there were no significant differences in baseline characteristics between the two groups (*P* > 0.05), including maternal age, BMI, duration of infertility, type of infertility, indications for IVF, antral follicle count, serum thyroid stimulating hormone (TSH), AMH, follicle-stimulating hormone (FSH), luteinizing hormone (LH), estradiol, progesterone, testosterone and prolactin levels. Additionally, the results indicated that the proportion of male fetuses in the PGT-A group was slightly higher than in the conventional IVF group. This difference may be due to the limitations of sample size or could suggest that PGT-A may have some influence on sex ratios, warranting further investigation.

**Table 1:** Baseline characteristics of the participants who achieved singleton live birth after PGT-A vs. conventional IVF.

Characteristics	PGT-A (*N* = 462)	Conventional IVF (*N* = 478)	*P* values
Age (years)	28.89 ± 3.53	29.18 ± 3.49	0.205
BMI (kg/m²)	23.00 ± 3.39	22.96 ± 3.45	0.873
Duration of infertility (years)	3.26 ± 2.03	3.44 ± 2.19	0.203
Type of infertility (n, %)			0.106
Primary	303 (65.58)	337 (70.50)	
Secondary	159 (34.42)	141 (29.50)	
Indication for IVF (n, %)			0.121
Ovulatory dysfunction	33 (7.14)	23 (4.81)	
Tubal factor	232 (50.22)	269 (56.28)	
Endometriosis	3 (0.65)	2 (0.42)	
Male factor	69 (14.94)	82 (17.15)	
Combined factors	97 (21.00)	84 (17.57)	
Unexplained	28 (6.06)	18 (3.77)	
Antral follicle count	22.24 ± 10.62	21.58 ± 9.71	0.321
TSH (μIU/mL)	2.24 ± 1.41	2.18 ± 1.22	0.542
AMH (ng/ml)	6.40 (4.14-10.18)	6.34 (4.18-9.67)	0.542
FSH (IU/L)	5.82 (4.99-6.84)	5.96 (5.11-6.77)	0.097
LH (IU/L)	5.51 (3.96-8.08)	5.71 (4.05-8.01)	0.670
Estradiol (pg/ml)	35.30 (26.83-46.04)	36.75 (27.85-49.01)	0.157
Progesterone (ng/ml)	0.40 (0.21-0.66)	0.40 (0.22-0.66)	0.972
Testosterone (ng/ml)	0.32 (0.22-0.45)	0.32 (0.24-0.46)	0.642
Prolactin (ng/ml)	15.87 (11.79-20.71)	16.22 (12.17-22.40)	0.319
Sex of fetuses (n, %) †			0.019
Male	267 (58.04)	241 (50.42)	
Female	193 (41.96)	237 (49.58)	

Data are presented as the mean ± standard deviation, median (interquartile range) or n (%). *P* < 0.05 was considered statistically significant. † Data were missing regarding fetal sex in 2 women in the PGT-A group. Abbreviations: PGT-A, preimplantation genetic testing for aneuploidy; IVF, *in vitro* fertilization; BMI, body mass index; TSH, thyroid stimulating hormone; AMH, anti-Mullerian hormone; FSH, follicle-stimulating hormone; LH, luteinizing hormone.

### Ovarian response and embryo quality

The outcomes of controlled ovarian hyperstimulation in the two groups are compared in **Table 2**. No significant differences were observed in the duration of ovarian stimulation, gonadotropin dose, serum estradiol, LH and progesterone levels on the hCG trigger day, endometrial thickness on the hCG trigger day, number of oocytes with diameters ≥ 14 mm and ≥ 18 mm on the hCG trigger day, number of retrieved, metaphase II (MII), and two pronuclei (2PN) oocytes, number of high-quality embryos on day 3 and number of high-quality embryos on day 5 or 6 (*P* > 0.05). This indicates that ovarian response and embryo quality were equivalent between the two groups.

**Table 2:** Comparison of COH outcomes of singleton pregnancy after PGT-A vs. conventional IVF.

Characteristics	PGT-A (*N* = 462)	Conventional IVF (*N* = 478)	*P* values
Duration of ovarian stimulation (days)	9.80 ± 1.88	9.88 ± 2.13	0.542
Gonadotropin dose (IU)	1493.75 (1171.88-1875.00)	1425.00 (1200.00-1950.00)	0.566
Estradiol level on HCG trigger day (pg/mL)	5667.50 (4108.50-7603.75)	5569.00 (4283.50-7271.50)	0.795
LH level on HCG trigger day (pg/mL)	1.99 (1.26-3.39)	2.05 (1.35-3.23)	0.926
Progesterone level on HCG trigger day (ng/L)	0.87 (0.59-1.23)	0.80 (0.53-1.17)	0.092
Endometrial thickness on HCG trigger day (mm)	10.65 ± 2.24	10.70 ± 2.18	0.696
≥14 mm oocytes on HCG trigger day	17.77 ± 5.40	17.09 ± 5.50	0.053
≥18 mm oocytes on HCG trigger day	7.75 ± 4.20	7.42 ± 4.11	0.218
No. of oocytes retrieved	20.31 ± 7.72	19.56 ± 6.65	0.112
No. of MII oocytes	17.34 ± 5.65	17.06 ± 5.33	0.436
No. of 2PN oocytes	13.60 ± 4.67	13.16 ± 4.40	0.133
No. of high-quality embryos on day 3	9.54 ± 4.32	9.30 ± 4.04	0.388
No. of high-quality embryos on day 5 or 6	7.36 ± 3.14	7.02 ± 2.82	0.086

Data are presented as the mean ± standard deviation or median (interquartile range). *P* < 0.05 was considered statistically significant. Abbreviations: PGT-A, preimplantation genetic testing for aneuploidy; IVF, *in vitro* fertilization; HCG, human chorionic gonadotropin; LH, luteinizing hormone; MII, metaphase II; 2PN, two pronuclei.

### Pregnancy complications

**Figure 2:**
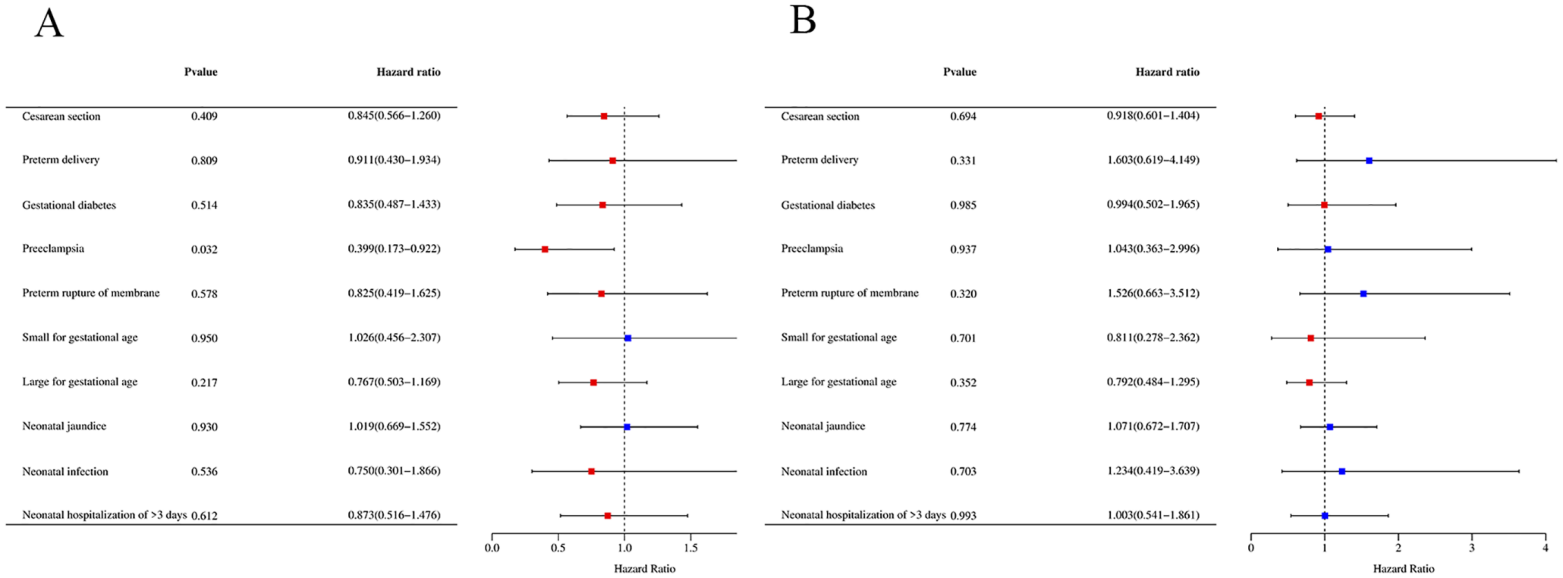
The risk of pregnancy complications based on fetal sex. **(A)** The risk of pregnancy complications in male fetuses. **(B)** The risk of pregnancy complications in female fetuses. OR > 1 indicates a risk factor, and OR < 1 indicates a protective factor.

Multivariable regression analyses showed no significant differences (*P* > 0.05) in the interaction between fetal sex and PGT-A vs. conventional IVF regarding risks of pregnancy complications, including gestational age at delivery, incidence of cesarean section, pre-term delivery, gestational diabetes, gestational hypertension, preeclampsia, placenta previa, or pre-term rupture of the membrane (**Table 3**). However, subgroup analysis based on fetal sex demonstrated a significantly lower risk of preeclampsia following PGT-A compared to conventional IVF in male fetuses (3.37% vs. 7.88%; adjusted OR, 0.40; 95% CI, 0.17-0.92; *P* = 0.032) (**Figure 2A** and **Supplementary Tables 1**, **2**). This protective effect was not observed in pregnancies with female fetuses (3.63% vs. 3.38%; adjusted OR, 1.04; 95% CI, 0.36-3.00; *P* = 0.937) (**Figure 2B**, **Supplementary Table 3**), suggesting that the impacts of PGT-A on preeclampsia incidence is dependent on fetal sex.

**Table 3:** Comparison of pregnancy complications of singleton pregnancy after PGT-A vs. conventional IVF.

Characteristics	PGT-A (*N* = 462)	Conventional IVF (*N* = 478)	Adjusted OR or absolute difference (95% CI)	*P* values for interaction between PGT-A and fetal sex
Gestational age at delivery (weeks)	39.21 ± 1.62	39.23 ± 1.48	0.02 (-0.38-0.42)	0.920
Male fetuses	39.11 ± 1.66	39.09 ± 1.56	-0.01 (-0.29-0.27)	
Female fetuses	39.36 ± 1.57	39.38 ± 1.37	-0.02 (-0.30-0.27)	
Cesarean section (n, %)	288 (64.29)	306 (66.67)	0.89 (0.49-1.58)	0.679
Male fetuses	171 (65.52)	159 (68.24)	0.85 (0.57-1.26)	
Female fetuses	117 (62.57)	147 (65.04)	0.92 (0.60-1.40)	
Preterm delivery (n, %)	27 (5.84)	23 (4.81)	0.51 (0.15-1.71)	0.278
Male fetuses	16 (5.99)	15 (6.22)	0.91 (0.43-1.93)	
Female fetuses	11 (5.70)	8 (3.38)	1.60 (0.62-4.15)	
Gestational diabetes (n, %)	47 (10.17)	54 (11.30)	0.81 (0.34-1.92)	0.630
Male fetuses	30 (11.24)	33 (13.69)	0.84 (0.49-1.43)	
Female fetuses	17 (8.81)	21 (8.86)	0.99 (0.50-1.97)	
Gestational hypertension (n, %)	10 (2.16)	7 (1.46)	3.29 (0.39-27.65)	0.272
Male fetuses	6 (2.25)	2 (0.83)	3.03 (0.59-15.48)	
Female fetuses	4 (2.07)	5 (2.11)	0.89 (0.22-3.57)	
Preeclampsia (n, %)	16 (3.46)	27 (5.65)	0.34 (0.09-1.29)	0.112
Male fetuses	9 (3.37)	19 (7.88)	0.40 (0.17-0.92)	
Female fetuses	7 (3.63)	8 (3.38)	1.04 (0.36-3.00)	
Placenta previa (n, %)	4 (0.87)	7 (1.46)	5.50 (0.32-94.02)	0.240
Male fetuses	3 (1.12)	2 (0.83)	1.78 (0.27-11.81)	
Female fetuses	1 (0.52)	5 (2.11)	0.28 (0.03-2.50)	
Preterm rupture of membrane (n, %)	33 (7.14)	31 (6.49)	0.54 (0.19-1.56)	0.252
Male fetuses	19 (7.12)	20 (8.30)	0.83 (0.42-1.63)	
Female fetuses	14 (7.25)	11 (4.64)	1.53 (0.66-3.51)	

Data are presented as the mean ± standard deviation, or n/N (%). *P* < 0.05 was considered statistically significant. The covariables in the overall regression models were maternal age, BMI, AMH, number of oocytes retrieved, number of high-quality embryos on day 5 or 6, endometrial thickness before embryo transfer, PGT-A vs. conventional IVF, fetal sex, and interaction between PGT-A vs. conventional IVF and fetal sex. For each fetal sex subgroup, the regression model included covariables of maternal age, BMI, number of oocytes retrieved, number of high-quality embryos on day 5 or 6, endometrial thickness before embryo transfer, and PGT-A vs. conventional IVF. Abbreviations: PGT-A, preimplantation genetic testing for aneuploidy; IVF, *in vitro* fertilization; OR, odds ratio; CI, confidence interval.

### Neonatal outcomes

As shown in **Table 4**, no significant differences were found in neonatal outcomes attributable to the interaction between fetal sex and PGT-A vs. conventional IVF (*P* > 0.05), including mean birth weight, incidence of SGA, LGA, neonatal respiratory distress syndrome (RDS), neonatal jaundice, neonatal infection and neonatal hospitalization of >3 days. For each fetal sex subgroup analysis, no significant difference in neonatal outcomes was detected between the PGT-A group and the conventional IVF group.

**Table 4:** Comparison of neonatal outcomes and birth weight of singleton pregnancy after PGT-A vs. conventional IVF.

Characteristics	PGT-A (*N* = 462)	Conventional IVF (*N* = 478)	Adjusted OR or absolute difference (95% CI)	*P* values for interaction between PGT-A and fetal sex
Mean birth weight (g)	3417.24 ± 487.71	3449.33 ± 488.19	-58.74 (-185.50-68.03)	0.363
Male fetuses	3440.79 ± 498.67	3506.49 ± 533.68	57.12 (-34.26-148.50)	
Female fetuses	3389.77 ± 471.68	3391.20 ± 430.52	0.27 (-86.32-86.87)	
SGA (n, %)	20 (4.35)	21 (4.39)	1.21 (0.32-4.59)	0.776
Male fetuses	14 (5.24)	12 (4.98)	1.03 (0.46-2.31)	
Female fetuses	6 (3.11)	9 (3.80)	0.81 (0.28-2.36)	
LGA (n, %)	91 (19.78)	114 (23.85)	0.95 (0.50-1.81)	0.876
Male fetuses	55 (20.60)	63 (26.14)	0.77 (0.50-1.17)	
Female fetuses	36 (18.65)	51 (21.52)	0.79 (0.48-1.30)	
Neonatal RDS (n, %)	3 (0.65)	5 (1.05)	0.09 (0.00-2.43)	0.153
Male fetuses	1 (0.37)	4 (1.66)	0.22 (0.02-2.02)	
Female fetuses	2 (1.04)	1 (0.42)	2.48 (0.20-30.06)	
Neonatal jaundice (n, %)	113 (24.46)	110 (23.01)	0.96 (0.51-1.78)	0.886
Male fetuses	65 (24.34)	57 (23.65)	1.02 (0.67-1.55)	
Female fetuses	48 (24.87)	53 (22.36)	1.07 (0.67-1.71)	
Neonatal infection (n, %)	16 (3.46)	18 (3.77)	0.56 (0.14-2.29)	0.423
Male fetuses	9 (3.37)	11 (4.56)	0.75 (0.30-1.87)	
Female fetuses	7 (3.63)	7 (2.95)	1.23 (0.42-3.64)	
Neonatal hospitalization of >3 days (n, %)	55 (11.90)	59 (12.34)	0.90 (0.40-2.01)	0.792
Male fetuses	34 (12.73)	33 (13.69)	0.87 (0.52-1.48)	
Female fetuses	21 (10.88)	26 (10.97)	1.00 (0.54-1.86)	

Data are presented as the mean ± standard deviation, or n/N (%). *P* < 0.05 was considered statistically significant. The covariables in the overall regression models were maternal age, BMI, AMH, number of oocytes retrieved, number of high-quality embryos on day 5 or 6, endometrial thickness before embryo transfer, PGT-A vs. conventional IVF, fetal sex, and interaction between PGT-A vs. conventional IVF and fetal sex. For each fetal sex subgroup, the regression model included covariables of maternal age, BMI, number of oocytes retrieved, number of high-quality embryos on day 5 or 6, endometrial thickness before embryo transfer, and PGT-A vs. conventional IVF. Abbreviations: PGT-A, preimplantation genetic testing for aneuploidy; IVF, *in vitro* fertilization; OR, odds ratio; CI, confidence interval; SGA, small for gestational age; LGA, large for gestational age; RDS, respiratory distress syndrome.

## Discussion

To our knowledge, no previous studies have examined the potential relationships between PGT-A and fetal sex in relation to the risk of pregnancy complications. In this *post hoc* exploratory secondary analysis of data from a multicenter randomized controlled trial, we provided evidence that PGT-A treatment may decrease the risk of preeclampsia in women carrying singleton male fetuses compared with those pregnant women who underwent conventional IVF treatment. Notably, this potential benefit of PGT-A treatment was not observed in mothers who delivered female fetuses. In addition, no obvious relationship between PGT-A and fetal sex in other pregnancy complications or neonatal outcomes, such as the mean birth weight, SGA, or LGA, was observed.

Given the unavoidable invasive nature of embryo biopsy during PGT-A, the choice of embryonic stage for biopsyis usually with cautious to minimize potential adverse impacts on embryo development. Current methods of PGT-A involve polar body biopsy (22), cleavage-stage biopsy (23), and blastocyst-stage biopsy (24). Among these, blastocyst-stage biopsy is most common due to its improved genetic testing accuracy and lesser impact on the inner cell mass. As the prevalence recognition of mosaic embryos which refer to the embryos possess two or more genetically different sets of cells, it has been realized that the biopsy and test of 5-10 trophectoderm cells cannot accurately reflect aneuploidy throughout the embryo and for the inner cell mass, let alone predicting pregnancy outcomes after embryo transfer (11, 25). Several studies have found that PGT-A can improve rates of clinical (3) and ongoing pregnancies (26), as well as effectively reduce the occurrence of adverse pregnancy outcomes such as pre-term birth and low birth weight (27). However, more recent randomized controlled studies have suggested that PGT-A does not improve ongoing pregnancy or live birth rates after frozen embryo transfer compared with conventional IVF treatment (28, 29). Our original randomized controlled trial also found that PGT-A does not improve the cumulative live birth rates after embryo transfer in patients with a good prognosis, except for the rate of first-trimester pregnancy loss, nor does it affect other pregnancy complications or neonatal outcomes (5). These inconsistent findings might be partially attributed to the self-correction ability of aneuploidy cells and the recognition of mosaic embryos as a normal stage in embryo development (30, 31).

While PGT-A may not accurately depict the chromosomal status of the inner cell mass, it could still theoretically aid in the prevention of diseases related to placental development, considering its ability to identify aneuploidy in the trophectoderm, which forms the placenta. Studies have demonstrated the presence of aneuploid cells in placentas of otherwise chromosomally normal pregnancies (32). It has been established that the placenta serves as a repository for unrepaired aneuploid cells, which can rectify embryonic endoderm aneuploidy, protect the fetus from aneuploidy, and promote implantation (10, 31). Notably, sex-based differences in disease risk, pathophysiology, clinical manifestations, and responses to clinical interventions have gained significant research attention recently (33). It has been indicated that the formation and function of the placenta are sex-dependent, with male fetuses having smaller placentas than female fetuses, suggesting an increased risk of adverse pregnancy events for male fetuses (12–14, 34). Recent studies suggests that the underlying mechanism of this observed phenomenon may be that the female placenta is more sensitive to abnormal intrauterine stress signals. This sensitivity could confer adaptive benefits through the inactivation of one X chromosome during preimplantation (35).

Abnormalities in the embryonic trophectoderm are known to be associated with the occurrence of pregnancy complications such as preeclampsia, which arises from impaired placental development (7). Given that the PGT-A during the trophectoderm development stage could potentially eliminate aneuploid cells that are resistant to self-correction later on, we hypothesize PGT-A could provide protective benefits against placental dysfunction-related pregnancy complications such as preeclampsia. Based on a secondary analysis of a large randomized controlled trial, our study provides evidence supporting the notion that PGT-A reduces the risk of maternal preeclampsia in singleton pregnancies carrying male fetuses compared to conventional IVF. It is important to note the differences in fetal sex between the PGT-A and conventional IVF groups, as these differences could influence the observed outcomes. While our findings suggest a potential protective effect of PGT-A, the mechanisms remain unclear, possibly relating to the timing or self-correction of mosaic cells in male versus female fetuses.

Ethical considerations surrounding sex selection in reproductive technologies, especially PGT-A, must be carefully examined. Choosing embryos based on sex can raise significant concerns about societal preferences for male or female offspring, potentially exacerbating gender imbalances. Additionally, while IVF is associated with an increased risk of preeclampsia, factors such as maternal age, underlying health conditions, and multiple gestations also play crucial roles (36). Currently, the proportion of male births during normal pregnancies in our country is relatively high (37), which may further influence these ethical dynamics, including the complex interplay of societal norms, reproductive choices, and potential long-term effects on gender ratios within the population. Further research is essential to disentangle these variables and comprehend the broader implications of our findings.

This study has several strengths, including the use of data from a prospective multicenter randomized controlled trial, which greatly minimizes potential bias compared to retrospective observational studies. We also have access to comprehensive data regarding baseline characteristics, as well as obstetric and neonatal outcomes. However, the robustness of our analysis was restricted by a relatively small sample size (N=940 singleton births), necessitating further larger studies. Furthermore, given that the original randomized controlled trial only enrolled women predicted to have a good prognosis for a live birth, whether our findings can be extended to patients with a predicted poor prognosis, such as those with chromosomal abnormalities that are indications for PGT-A treatment, needs further evaluation. Additionally, as the original study focused on cumulative live birth rates, the available information on pregnancy complications, particularly preeclampsia, is quite limited. Future research could explore detailed clinical characteristics related to preeclampsia for a more comprehensive understanding.

In conclusion, our study demonstrates that the sex of the fetus significantly influences the maternal risk of preeclampsia in singleton pregnancies following PGT-A treatment as compared to conventional IVF treatment. Specifically, pregnant women carrying male fetuses are likely to experience a decreased risk of preeclampsia after undergoing PGT-A treatment compared to those who received conventional IVF treatment. In this sense, our findings suggest a potential value in considering the sex-dependent influence of PGT-A treatment on the pathogenesis of preeclampsia, beyond its known protective role in reducing the rate of spontaneous abortion. In the future, additional large cohort studies are needed to not only reaffirm our findings but also to uncover the possible underlying mechanisms for these observed correlations.

## Data Availability

The raw data supporting the conclusions of this article will be made available by the authors, without undue reservation.
