# Supplementary material for: Comparative study on pregnancy complications: PGT-A vs. IVF-ET with gender-specific outcomes
Source: Front Endocrinol (Lausanne). 2024 Nov 6;15:1453083. doi: 10.3389/fendo.2024.1453083 (PMC11579861; doi:10.3389/fendo.2024.1453083)
Supplement: Supplementary file 1 [file Table1.docx]

Supplementary Material

# Supplementary Tables

**Supplementary Table 1. Results of multivariable logistic regression analysis for the risk of preeclampsia**

| Variables | Adjusted odds ratio and 95% CI | *P* value |
| --- | --- | --- |
| Age | 1.03 (0.94-1.13) | 0.483 |
| BMI | 1.14 (1.05-1.23) | 0.002* |
| AMH | 0.96 (0.89-1.04) | 0.348 |
| No. of oocytes retrieved | 1.02 (0.97-1.07) | 0.521 |
| No. of high-quality embryos on day 5 or 6 | 0.91 (0.80-1.03) | 0.129 |
| Endometrial thickness before embryo transfer | 0.08 (0.01-0.64) | 0.018* |
| PGT-A vs. Conventional IVF | 1.15 (0.41-3.28) | 0.789 |
| Fetal sex | 2.56 (1.09-6.04) | 0.032* |
| Interaction between PGT-A and fetal sex | 0.34 (0.09-1.29) | 0.112 |
| Constant | 0.01 | 0.034* |

*P* < 0.05 was considered statistically significant and indicated by an asterisk. The covariables in the regression models were maternal age, BMI, AMH, number of oocytes retrieved, number of high-quality embryos on day 5 or 6, endometrial thickness before embryo transfer, PGT-A vs. conventional IVF, fetal sex, and interaction between PGT-A vs. conventional IVF and fetal sex. Abbreviations: CI, confidence interval; BMI, body mass index; AMH, anti-Mullerian hormone; PGT-A, preimplantation genetic testing for aneuploidy; IVF, *in vitro* fertilization.

**Supplementary Table 2. Results of multivariable logistic regression analysis for the risk of preeclampsia in male fetuses**

| Variables | Adjusted odds ratio and 95% CI | *P* value |
| --- | --- | --- |
| Age | 1.07 (0.96-1.19) | 0.236 |
| BMI | 1.18 (1.07-1.30) | 0.001* |
| AMH | 0.94 (0.84-1.05) | 0.251 |
| No. of oocytes retrieved | 1.02 (0.96-1.09) | 0.479 |
| No. of high-quality embryos on day 5 or 6 | 0.86 (0.73-1.02) | 0.089 |
| Endometrial thickness before embryo transfer | 0.25 (0.02-3.51) | 0.302 |
| PGT-A vs. Conventional IVF | 0.40 (0.17-0.92) | 0.032* |
| Constant | 0.00 | 0.015* |

*P* < 0.05 was considered statistically significant and indicated by an asterisk. The covariables in the regression models were maternal age, BMI, AMH, number of oocytes retrieved, number of high-quality embryos on day 5 or 6, endometrial thickness before embryo transfer, and PGT-A vs. conventional IVF. Abbreviations: CI, confidence interval; BMI, body mass index; AMH, anti-Mullerian hormone; PGT-A, preimplantation genetic testing for aneuploidy; IVF, *in vitro* fertilization.

**Supplementary Table 3. Results of multivariable logistic regression analysis for the risk of preeclampsia in female fetuses**

| Variables | Adjusted odds ratio and 95% CI | *P* value |
| --- | --- | --- |
| Age | 0.94 (0.80-1.10) | 0.427 |
| BMI | 1.07 (0.92-1.25) | 0.372 |
| AMH | 0.99 (0.89-1.12) | 0.983 |
| No. of oocytes retrieved | 1.00 (0.92-1.08) | 0.988 |
| No. of high-quality embryos on day 5 or 6 | 0.96 (0.78-1.18) | 0.693 |
| Endometrial thickness before embryo transfer | 0.01 (0.00-0.46) | 0.019* |
| PGT-A vs. Conventional IVF | 1.04 (0.36-3.00) | 0.937 |
| Constant | 4.69 | 0.670 |

*P* < 0.05 was considered statistically significant and indicated by an asterisk. The covariables in the regression models were maternal age, BMI, AMH, number of oocytes retrieved, number of high-quality embryos on day 5 or 6, endometrial thickness before embryo transfer, and PGT-A vs. conventional IVF. Abbreviations: CI, confidence interval; BMI, body mass index; AMH, anti-Mullerian hormone; PGT-A, preimplantation genetic testing for aneuploidy; IVF, *in vitro* fertilization.
